# The rhesus macaque is three times as diverse but more closely equivalent in damaging coding variation as compared to the human

**DOI:** 10.1186/1471-2156-13-52

**Published:** 2012-06-29

**Authors:** Qiaoping Yuan, Zhifeng Zhou, Stephen G Lindell, J Dee Higley, Betsy Ferguson, Robert C Thompson, Juan F Lopez, Stephen J Suomi, Basel Baghal, Maggie Baker, Deborah C Mash, Christina S Barr, David Goldman

**Affiliations:** 1 Laboratory of Neurogenetics, National Institute on Alcohol Abuse and Alcoholism, NIH, Bethesda, MD, 20892, USA; 2 Laboratory of Clinical and Translational Studies, NIAAA, Bethesda, MD, 20892, USA; 3 Oregon National Primate Research Center, Oregon Health and Sciences University, 505 NW 185th Ave, Beaverton, OR, 97006, USA; 4 Department of Psychiatry, University of Michigan, Ann Arbor, MI, 48104, USA; 5 Mental Health Research Institute, University of Michigan Medical Center, 3064 NSL, 1103 East Huron Street, Ann Arbor, MI, 48104, USA; 6 Laboratory of Comparative Ethology, National Institute of Child Health and Human Development, NIH, Poolesville, MD, 20837, USA; 7 Department of Neurology, University of Miami School of Medicine, Miami, FL, 33136, USA

**Keywords:** Rhesus macaque, Human, Single nucleotide polymorphism, Diversity, Comparative genomics

## Abstract

**Background:**

As a model organism in biomedicine, the rhesus macaque (*Macaca mulatta*) is the most widely used nonhuman primate. Although a draft genome sequence was completed in 2007, there has been no systematic genome-wide comparison of genetic variation of this species to humans. Comparative analysis of functional and nonfunctional diversity in this highly abundant and adaptable non-human primate could inform its use as a model for human biology, and could reveal how variation in population history and size alters patterns and levels of sequence variation in primates.

**Results:**

We sequenced the mRNA transcriptome and H3K4me3-marked DNA regions in hippocampus from 14 humans and 14 rhesus macaques. Using equivalent methodology and sampling spaces, we identified 462,802 macaque SNPs, most of which were novel and disproportionately located in the functionally important genomic regions we had targeted in the sequencing. At least one SNP was identified in each of 16,797 annotated macaque genes. Accuracy of macaque SNP identification was conservatively estimated to be >90%. Comparative analyses using SNPs equivalently identified in the two species revealed that rhesus macaque has approximately three times higher SNP density and average nucleotide diversity as compared to the human. Based on this level of diversity, the effective population size of the rhesus macaque is approximately 80,000 which contrasts with an effective population size of less than 10,000 for humans. Across five categories of genomic regions, intergenic regions had the highest SNP density and average nucleotide diversity and CDS (coding sequences) the lowest, in both humans and macaques. Although there are more coding SNPs (cSNPs) per individual in macaques than in humans, the ratio of d_N_/d_S_ is significantly lower in the macaque. Furthermore, the number of damaging nonsynonymous cSNPs (have damaging effects on protein functions from PolyPhen-2 prediction) in the macaque is more closely equivalent to that of the human.

**Conclusions:**

This large panel of newly identified macaque SNPs enriched for functionally significant regions considerably expands our knowledge of genetic variation in the rhesus macaque. Comparative analysis reveals that this widespread, highly adaptable species is approximately three times as diverse as the human but more closely equivalent in damaging variation.

## Background

The rhesus macaque (*Macaca mulatta*) and human (*Homo sapiens*) are thought to have shared a common ancestor approximately 25 million years ago [1]. Due to their genetic, physiological and behavioral similarities with humans, and because of their hardiness, adaptability, and availability, the rhesus macaque has been widely used as a model in biomedical research [2,3]. Humans presently are the most numerous and widespread of primates. Furthermore hominid apes representing the ancestral lineage of humans were geographically widespread, their fossils having been found in both Africa and Asia. However the human diaspora is relatively recent, with our African ancestry dating back only 80,000 to 150,000 yrs b.p [4]. Also, the number of humans worldwide numbered as low as one million as recently as 100,000 yrs ago [5], and due to limitations in dispersion and gene flow effective population sizes were much smaller still. Substantial evidence exists that the neutral genetic diversity of humans has been shaped, and in fact restricted, by an effective population size that until recently was less than 8,000 [6]. Current geographic range of the rhesus macaque extends from Afghanistan to the East China Sea. The population presently numbers in the millions, and in its range and population size the rhesus macaque is only exceeded by the humans among primate species [7]. Fossil evidence indicates that the *Macaca* genus originated in North Africa, and dispersed to various sites in Asia at least three million years ago [8]. The rhesus macaque has adapted to a variety of natural environments, including savannah and forests, and has adapted to various climatic zones. Rhesus macaques thrive in cities where they live side by side with man. The diversity of environmental adaptations and large current and ancestral population sizes suggests that the genetic legacy of the rhesus macaque may include a higher quotient of both neutral and selectively significant genetic variation than humans. Understanding the details of differences in genomic diversity between macaques and humans, especially in functionally important genomic regions, will not only provide valuable information on their evolutionary dynamics but improve the utility of the rhesus macaque as a non-human primate model in biomedical research.

The rhesus macaque is a genetically diverse primate. Consistent with the rhesus macaque having a high degree of genetic variation, substantial morphological variation has been observed between rhesus macaques from the same populations and also between populations, with as many as 13 subspecies identified [9]. Within rhesus macaques there is some evidence for genetic distinctiveness at the molecular level, and Indian rhesus may be among the least diverse [10]. Several studies using protein polymorphisms have found higher levels of diversity in rhesus macaques from China (where there are also more subspecies) than India, and there is some evidence for a genetic bottleneck in Indian rhesus macaques [9]. However, substantial gene flow probably occurred later, which could refresh genetic variation. In a study of six rhesus macaque populations, including Indian, Burmese, and four Chinese populations, Indian macaques had one third to one sixth the mitochondrial DNA diversity as compared to four other populations. However, Indian macaques were approximately equal in diversity to rhesus macaques from one of the Western Chinese populations [9]. A recent study with more than 1,000 Single nucleotide polymorphisms (SNPs), which are more mutationally stable than other types of genetic markers, revealed that Indian and Chinese rhesus macaques were nearly identical in genetic diversity [11]. Taken together, the evidence suggests that the rhesus macaque is likely to be a genetically diverse primate species but Indian macaques are if anything among the least heterogeneous populations. Genomic analysis of rhesus macaques of Indian origin, which are more often used for biomedical research, would thus provide a conservative estimate of the variability of rhesus macaques.

A single rhesus macaque of Indian origin was the source for a macaque draft genome sequence, which was completed in 2007 [3]. This draft sequence opened the opportunity to map the amount and type of macaque genomic variation. Furthermore, characterization of genetic variation in macaques would greatly improve the value of the rhesus macaque as an animal model for biomedical research and human biology. However, just 8,134 macaque SNPs have currently been recorded in dbSNP (Build 135**,**http://www.ncbi.nlm.nih.gov/SNP/snp_summary.cgi). In 2007, Malhi et al. reported approximately 23,000 candidate SNPs detected by pyrosequencing [12]. Fawcett et al. recently reported 3 million SNPs in Indian-origin rhesus macaques using SOLiD re-sequencing along with previous sequencing data [13]. Some 22 million SNPs are known in the human. The number of SNPs in the macaque is unknown, but may be much larger. Based on their observation of 3 million SNPs, Fawcett et al. suggested that the rhesus macaque is at least as diverse as the human, or more diverse, but did not analyze the two species with an equivalent approach and refrained from a direct quantitative comparison, as we will perform here.

There have been some more limited efforts to comparatively estimate diversity in the rhesus macaque. As revealed in the original sequencing of a single animal and compatible with a larger effective population size of the macaque across evolutionary timeframes, the macaque appeared to have higher sequence diversity than the human [3,14]. SNP density was broadly estimated to range between 1–7.8 SNPs/Kb [3,15]**.** However, the number of loci on which this conclusion was based was relatively small, and the loci were not selected in unbiased fashion. In this study, we have used SNPs equivalently identified in 14 humans and 14 rhesus macaques (mostly of Indian origin) by massively parallel sequencing with both H3K4me3 (trimethylated histone H3-lysine 4) ChIPseq (chromatin immunoprecipitation followed with massively parallel DNA sequencing) and RNAseq (whole transcriptome massively parallel shotgun sequencing) as sources of sequenced fragments. From more than 16,000 genic regions, some half million macaque SNPs, most newly identified, were further analyzed. By sequencing diversity in the tissue-specific transcriptomes and histone-marked regions of the two species, we were able, without the use of DNA capture technology (which did not exist for the macaque) or whole-genome sequencing, to compare diversity in equivalent, functionally relevant regions and detect effects of selection and drift on sequence substitutions.

## Results and discussion

### SNP density is three times higher in the rhesus macaque than the human

Diversity was determined in short sequence reads (36 bases) equivalently detected and analyzed in 14 humans and 14 rhesus macaques (Table 1). All raw sequences generated in this study have been deposited in the Sequence Read Archive (NCBI) with the accession numbers SRA028822, SRA027316, SRA029279 and SRA029275. It is important to point out that the analytical strategy of comparing diversity within the hippocampal transcriptome and in H3K4me3-marked DNA regions of chromatin from hippocampus resulted in the analysis of equivalent genomic regions in the macaque and in the human. There was a strong correlation between level of expression of genic associated sequences between the hippocampus of both species and in the regions strongly tagged by H3K4me3 (Additional file 1 Figure S1). From these equivalent genomic regions with at least 3x sequencing coverage, a total of 462,802 high quality putative SNPs (most of which were novel) were detected in the macaque, and 230,028 SNPs (most of which were known) were detected in the human. At least one SNP was identified in 14,675 human annotated genes and 16,797 macaque annotated genes. Approximately 10-25% of the putative SNPs detected in intergenic regions were covered with RNAseq reads (Additional file 1 Table S2), suggesting that significant transcriptional activity occurred outside of defined genic regions in both species, consistent with other reports [16]. Among 230,028 putative human SNPs, 90% had been recorded previously in dbSNP. This rediscovery rate is slightly higher than the 77-89% rediscovery rate for SNPs in the 1000 Genomes Project Pilot 2 deep sequencing (Additional file 1 Table S3) [17]. Also bearing on the validity of the SNP detection pipeline, the transition/transversion ratio of human and macaque SNPs was non-random. Although the random transition/transversion ratio is 1:2, this ratio was approximately 2:1 in both species. 22 of 26 human nsSNPs detected using the same SNP detection pipeline were validated by Sanger sequencing in a previous study [18]. In this study, 13 of 14 novel macaque cSNPs were verified by Sanger sequencing. The chromosome positions and alleles of all SNPs discovered in this study can be accessed at: http://pubs.niaaa.nih.gov/publications/LNGBMCGenetics2012/LNG_2012.htm.

**Table 1:** **Sequence coverage and putative SNPs detected in 14 rhesus macaques (*Macaca mulatta*) and 14 humans (*Homo sapiens*) using parallel methods in a genic-enriched target region**

		**Human**	**Macaque**
Genome size in reference assembly (Mb)		3,080	2,864
Non-gap reference genome size (Mb)		2,858	2,647
Unique coding sequence size in reference (Mb)		32.5	31.8
Sample number		14	14
Average 36-base reads per sample		17.4 x 10^6^	14.4 x 10^6^
Total length (Mb) of uniquely mapped reads		8,770	7,266
Mb in genome with (≥1x sequence coverage)		1,505	1,571
Mb in genome with (≥3x sequence coverage)		426	435
SNPs in dbSNP_B 131		23,653,737	7,880
SNPs in this study		230,028	462,802
Also in dbSNP_B131		206,267(89.7%)	34(0.0%)
Transition	AG,GA,TC,CT	155,836(67.7%)	312,643(67.4%)
Transversion	AC,CA,TG,GT	37,046(16.1%)	79,061(17.1%)
Transversion	CG,GC	25,467(11.1%)	46,820(10.1%)
Transversion	AT,TA	11,679(5.1%)	24,857(5.4%)
Genes with SNPs		14,675	16,797
Genes with SNPs in exons		11,200	12,466
SNPs located in intergenic regions		107,461(46.7%)	269,390(58.2%)
SNPs located in 5Kb upstream of TSS		10,036(4.4%)	26,303(5.7%)
SNPs located in UTR		18,432(8.0%)	15,455(3.3%)
SNPs located in intron		79,875(34.7%)	130,443(28.2%)
SNPs located in CDS		14,224(6.2%)	21,211(4.6%)
Synonymous		8,329(58.6%)	13,798(65.1%)
Non-synonymous		5,877(41.3%)	7,367(34.7%)
Damaging*		1,741 (29.6%)	1,525 (20.7%)
Nonsense		18(0.1%)	46(0.2%)

*damaging as evaluated by Polyphen.

**Figure 1:**
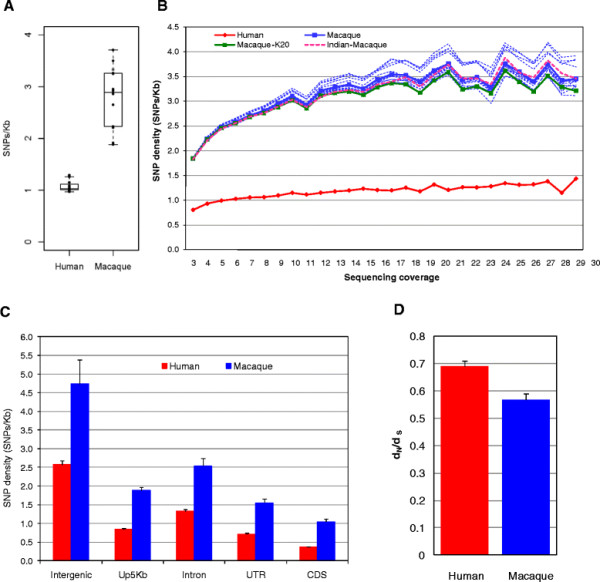
**Average SNP density (SNPs/Kb) in humans and rhesus macaques, comparatively measuring variation in different genomic regions.A**). SNPs/Kb is calculated for nucleotides with > = 4x sequence coverage. **B**). Average SNP density of human and macaque calculated for different sequencing coverages. SNP density based on all macaque samples is shown with the blue line. SNP density with macaque K20 omitted is shown with the green line and SNP densities with other individual animals omitted one-by-one are shown with dashed blue lines. SNP density based on 100% Indian macaques only is shown with the pink dashed line. **C**). The SNP densities of five genomic regions in human and macaque; **D**). Human and macaque d_N_/d_S_ ratios for cSNPs. Error bars in (C) and (D) indicate standard error of mean.

Overall, the rhesus macaque had a SNP density approximately three times higher than humans (Figure 1A). Calculated across all genomic regions with at least 4x sequencing coverage in individual samples, the SNP densities for macaques and humans were 2.82 SNP/kb and 1.07 SNP/Kb, respectively (Table 2, Figure 1A`). Because sequencing coverage for individual samples was low for most regions, putative SNPs were called by a conservative, two-step approach as described in methods. As a result, SNP density increased in both species as sequencing coverage increased (Figure 1B), but it can be observed that the macaque had proportionately higher SNP densities at all levels of sequencing coverage (Figure 1B). One of the macaque samples was of Chinese origin and two were approximately equally admixed between Chinese macaque and Indian macaques as described in methods. However, in the comparison between macaque and human, this Chinese macaque (K20) and the two admixed macaques did not exert a larger effect on SNP density as compared to any of the Indian macaques. This was tested by omitting individual macaques one-by-one, and also by evaluating SNP density with all three of the animals with any Chinese ancestry omitted (Figure 1B). The result is consistent with a recent study which found no difference in genetic diversity between Chinese and Indian macaques via analyses with more than 1,000 SNPs [11]. As mentioned, our human sample itself included individuals of different ethnic backgrounds. Therefore, the Chinese macaque and the two admixed animals were included in all analyses unless specified otherwise.

**Table 2:** Comparative SNP density based on sequencing studies of the human and the rhesus macaque

**Genome**	**Technology used**	**SNPs/Kb**
Venter	Sanger method	1.41*
Watson	454 Sequencing System (Roche)	1.46*
Chinese (YH)	Genome Analyzer (Illumina)	1.35*
African (NA18507)	Genome Analyzer (Illumina)	1.58*
African (NA18507)	SOLiD system (Applied Biosystems)	1.69*
Korean (SJK)	Genome Analyzer (Illumina)	1.50*
Korean (AK1)	Genome Analyzer (Illumina)	1.51*
Proband (III-4)	SOLiD system (Applied Biosystems)	1.50*
CEU,YRI	Genome Analyzer, SOLiD, 454	1.21-1.48**
Humans, in this study	Genome Analyzer (Illumina)	1.07(0.97-1.26)
Macaques, in this study	Genome Analyzer (Illumina)	2.82(1.88-3.71)

*The SNP number was from Lupski et al. 2010 [19] and SNPs/Kb was calculated based on the total SNPs reported, an estimated size of 2.85 x 10^9^ for the sequenced human genome and an estimate that 80% of the genome was accessible, for example due to mappability [17].

**Based on SNPs detected and accessible genome size from the high coverage Pilot Trios data of the 1000 Genomes Project [17].

At higher coverage, the SNP density we detected in humans closely approached that found by highest coverage sequencing, being 1.5 SNPs/Kb for 30x coverage across 14 human samples. A range of 3.07 ~ 3.86 x 10^6^ SNPs was reported for individual human genomes [19] representing approximately 1.3 ~ 1.7 SNP/Kb. Also, a SNP density of 1.2 ~ 1.5 SNPs/Kb can be derived from the 1000 Genomes Project Pilot 2 data for two human family trios with >40x sequencing coverage [17]. In our study, SNP densities were estimated from 14 samples in both species and with highly similar sequencing coverage, representing a methodologically equivalent view of diversity. Since intergenic and intronic regions comprise the majority of the genome in both humans and macaques, the overall SNP densities reported here are most likely underestimates because a high proportion of our data derives from coding sequences (CDS) and untranslated regions (UTR) that have the lowest SNP densities, as will be discussed below and as shown in Figure 1C.

We compared SNP density across five different categories of genomic regions: intergenic, 5 Kb upstream of TSS (transcription start site), introns, UTR (5’- and 3’-UTRs), and CDS as annotated in refGene (human) or ENSEMBL (macaque). In all five genomic regions, macaques had significant (p-value < 0.0001 by Wilcoxon rank sum test in each genomic region) higher SNP densities than humans (Figure 1C). Intergenic regions had the highest SNP density and coding regions the lowest SNP density in both species (Figure 1C). In coding regions, 76% of the cSNPs would be expected to be nsSNPs if all base substitutions were equally likely [20]. However, nsSNP density was lower than synonymous cSNP density with a d_N_/d_S_ ratio **(**ratio of nonsynonymous to synonymous substitutions, reflecting selection pressure acting on nonsynonymous sites relative to synonymous ones) in humans of approximately 0.691 ± 0.017 and a d_N_/d_S_ ratio of 0.567 ± 0.022 in macaque (Figure 1D). Although both adaptation and purifying selection may have occurred at numerous genes in both species, purifying selection is most likely to be predominant across the whole genome in both species as their d_N_/d_S_ ratio values are significantly less than 1. In the recently reported analysis by Fawcett et al., the macaque d_N_/d_S_ ratio was close to 1, which would represent a significant departure from what has been observed in the human. The reasons for this are unclear, but based on our data where we have found similar d_N_/d_S_ ratios for the two species with equivalent methodology, we suggest that the macaque is similar to the human in also exhibiting evidence for adaptation and purifying selection at many genes, and despite other differences in overall variation that will be discussed in later sections.

Selection pressure on nonsynonymous substitutions may have been stronger in the macaque than in the human because the d_N_/d_S_ ratio in macaque is significantly (p-value <0.01 by Wilcoxon rank sum test) lower than human. In an equivalent genomic search space, twice as many putative SNPs were identified in macaque as compared to the human (Table 1). However, macaques only had 1.2 times as many nsSNPs, indicating that much of the increased diversity of the macaque, even in protein-coding regions of the genome, is likely to be selectively neutral. Furthermore, the nsSNPs of macaques were less likely to be damaging (predicted to have damaging effects on protein functions by PolyPhen-2, including “possibly damaging” and “probably damaging”) as compared to the human (20% in macaque vs 30% in human) (Table 1). In line with this result, the higher d_N_/d_S_ ratio in human may reflect a relative relaxation of purifying selection during hominoid evolution as a consequence of smaller effective population sizes or a high rate of adaptive substitution [21].

Using RNAseq and H3K4me3 ChIPseq data, a relatively high percentage of SNPs is identified in gene coding and promoter regions, which represent functionally important domains of the genome. This could represent an advantage for certain types of gene-centric analyses. For instance, 6.2% of the human SNPs detected in this study (90% of which were previously known) were located in coding regions (cSNPs), whereas only 0.7% of the total SNPs identified in 1000 Genomes Project Pilot 2 data were cSNPs (Additional file 1 Table S3) [17]. Here we sequenced only 0.426 Gb of unique human sequence at ≥3x coverage, but detected 14,224 cSNPs. This is a substantial number given that 24,192 cSNPs were detected in three Caucasian individuals whose genomes were sequenced at high coverage, in the 1000 Genomes Project Pilot 2 (Additional file 1 Figure S2). While only 0.3% of the 3 million most recent reported macaque SNPs were in coding regions [13], 4.6% of the macaque SNPs in this study are cSNPs (Table 1). The major limitation for SNP detection here was the proportion of genes that are not expressed in adult hippocampus or that are expressed at a low level in this tissue due to age, sex, or tissue specific expression. The overlap of the cSNPs we detected with those reported in two individuals from the 1000 Genomes Project Pilot 2 data is consistent with the overlap that has been empirically observed between unrelated individuals (50 ~ 70% SNPs shared) on a pair-wise basis (Additional file 1 Figure S3) [17]. Based on our sensitivity of detection of human SNPs, where 14,224 cSNPs were detected versus some 250,000 cSNPs that have been reported in NCBI (from a much larger population of subjects), we estimate that our region-focused sequencing of only 14 individuals enabled us to discover approximately 6% of the common cSNPs that are present in the rhesus macaque, although detection sensitivity was of course higher for the more abundant SNPs, and this greatly underestimates the number of rare and uncommon sequence variants possessed by the rhesus macaque.

### Rhesus macaques are three times as diverse as the human

**Figure 2:**
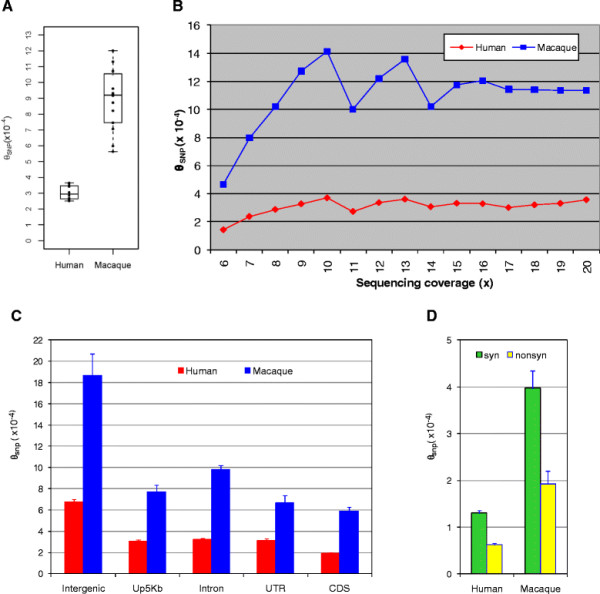
**Average nucleotide diversity (θ**_**SNP**_**) of humans and rhesus macaques, comparatively measuring variation in different genomic regions. A).** θ_SNP_ is calculated for nucleotides with > = 6x sequence coverage. **B**). Nucleotide diversity of human and macaque calculated for different sequencing coverages. **C**). Average nucleotide diversity of five different genomic regions; **D**). Average nucleotide diversity for synonymous cSNPs and nsSNPs. Error bars in (C) and (D) indicate standard error of mean.

The average nucleotide heterozygosity (diversity) for SNPs (θ_SNP,_ as defined by Levy et al. 2007 [22]) was measured as the ratio of heterozygous basepairs (both alleles with ≥3x coverage and ≥ 30% of sequence reads) divided by all basepairs sequenced at this level, within each individual. Macaque θ_SNP_ was 3 times higher than human θ_SNP_ (8.93x10^-4^ vs 3.06x10^-4^, Figure 2A). Paralleling observations on SNP density, as sequencing coverage increased, more heterozygous basepairs were detected. With increasing sequencing coverage, θ_SNP_ increased, becoming asymptotic at about 10x coverage (Figure 2B). At 20x sequencing coverage, θ_SNP_ was 11.4 x 10^-4^ in the macaque and 3.6 x 10^-4^ in the human (Figure 2B). Similar to what we observed for SNP density, θ_SNP_ was highest in intergenic regions and lowest in coding regions in both species and macaque had significant higher θ_SNP_ than human in all five genomic regions (Figure 2C). Our estimated θ_SNP_ in human from all regions (3.06 × 10^−4^) is somewhat lower than values that can be calculated (Additional file 1 Table S4) from 1000 Genomes Project Pilot 2 data (7.2 × 10^−4^ to 9.3 × 10^−4^) and is also slightly lower than values of 5.4 × 10^−4^ to 8.3 × 10^−4^ reported previously for humans [22-26]. Our overall lower human θ_SNP_ as compared to previously reported values was expected due to the higher genic percentage of sequenced regions. In line with this assertion, within intergenic regions that comprise most of the genome our θ_SNP_ estimate of ~6.78 x 10^−4^ is very close to the previous estimates based on high coverage whole genome sequencing. Therefore, although we do not have a direct reference sample for the macaque, the θ_SNP_ values we have computed for both macaque and human appear to be robust, reflect parallel methodology and sampling and are informative for both genome-wide and regional increases in genetic diversity in the macaque compared to human.

**Figure 3:**
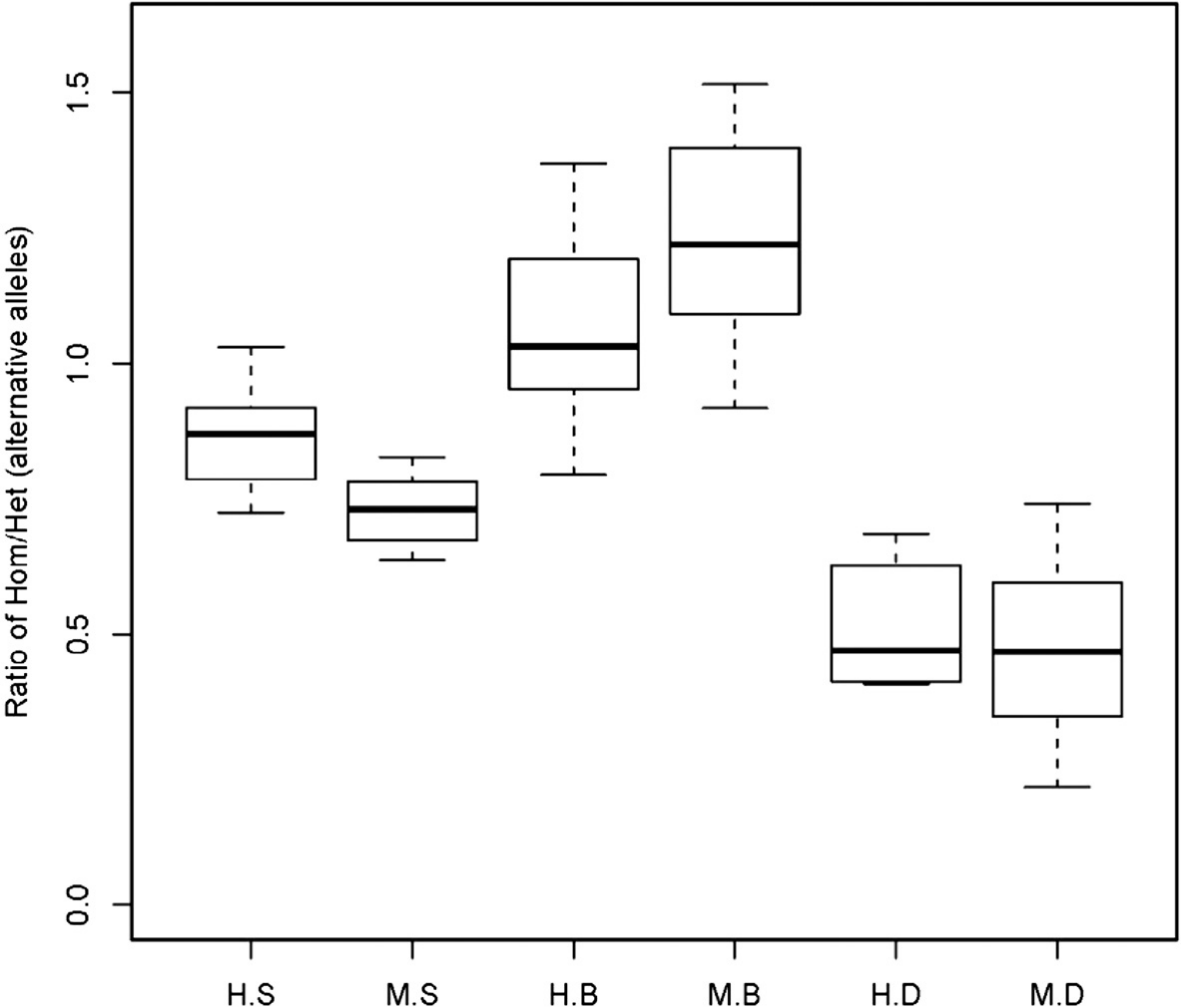
**Analysis of homozygosity/heterozygosity for cSNPs, based on the idea that severely damaging variants are less likely to be homozygous.** H.S: human synonymous cSNPs; M.S: macaque synonymous cSNPs; H.B: human nsSNPs predicted to be “benign” by PolyPhen; M.B: macaque nsSNPs predicted to be “benign” by PolyPhen; H.D: human nsSNPs predicted to be damaging by PolyPhen; M.D: macaque nsSNPs predicted to be damaging by PolyPhen.

Within coding regions it is possible to compare diversity that is more likely to be functionally significant with diversity that is more likely to be selectively neutral. In coding regions, both human and macaque had approximately 2 times more diversity for synonymous cSNPs as compared to nsSNPs (Figure 2D), reflecting the effects of functional constraints and selection against changes in protein sequence [25]. Concerning the possible functional significance of nsSNPs, Polyphen predicted that some 1,741 (29.6%) human cSNPs and 1,525 (20.7%) macaque cSNPs were likely to be damaging. Fawcett et al. also reported a similar ratio for damaging macaque cSNPs, finding that 17.6% of 4,177 nsSNPs were damaging, and they also used the same program, Polyphen [13]. The macaque cSNPs we identified include a substantial resource of putatively functional sequence variants, adding to ones detected by Fawcett et al. Supporting the functional significance of many of these SNPs, individual humans and macaques were both half as likely to be homozygous for damaging nsSNPs than they were to be homozygous for synonymous cSNPs and nsSNPs scored as benign (Figure 3).

In line with theory that most of the increased diversity of the rhesus macaque should be selectively neutral in nature, the increase in macaque SNP density was not proportionately maintained from non-coding sequence to coding sequence, to nsSNPs and to putatively damaging nsSNPs. Instead, the macaque more closely resembled the human in its SNP density within these more functionally significant categories. However, a different picture was observed using the diversity measure θ_SNP_. By this standard, the macaque was approximately three times as diverse as the human across all types of sequence categories. This could point to the maintenance of nsSNPs by balancing selection. This is an important mechanism of evolutionary adaptation in all genetically diverse species but may be operative at a larger percentage of loci in the macaque than in the human. Speculatively, although the macaque does not have proportionately more nsSNPs, it may be that those that it does have are more likely to be maintained at higher frequency by balancing selection. Although this would explain the reason for which nsSNP density does not increase proportionately with overall SNP density and with diversity, other validating data would be required to establish this point. One indirect test would be linkage disequilibrium analysis that could detect signals of selection (selective sweeps) at genes containing nsSNPs. In fact, one use of the SNPs we and Fawcett et al. have discovered would be the creation of marker panels enabling genome wide evaluation of LD. When that is done, the results may again be surprising.

At equilibrium, LD depends on the recombination rate and effective population size. Therefore, it might be anticipated that LD blocks in the rhesus macaque will be substantially smaller than the human. Thus a macaque SNP panel effective for genome-wide use might have to be larger than human 1 M panels that are now the standard. However, it is also possible that cross-population admixture has already occurred in the rhesus macaque, at least in some samples of macaques, which could have led to the presence of much larger haplotype blocks than anticipated on the basis of population size. For example, LD blocks in some Indian macaques were observed to be even larger than humans [15]. On average Chinese macaques have lower LD with a greater proportion of rare alleles than Indian macaques [15,27]. Differences in linkage disequilibrium and diversity between populations of rhesus macaque could be useful in biomedicine, for example to close in on the identity of functional loci altering traits. The macaques analyzed here are primarily of Indian origin. Our findings on the relative diversity of Chinese and Indian macaques were limited because we studied only one individual animal of Chinese origin and two that were admixed. Furthermore, the specific geographic origin of this one Chinese macaque and the admixture component of the two other macaques, was unknown. That could be relevant, because the mitochondrial diversity of rhesus macaques from one Western Chinese population appeared to be equivalent to Indian macaques [9], which displayed lower mitochondrial diversity than several other macaque populations. However, it should be noted that the genetic diversity of nuclear DNA is less sensitive to the effects of population bottlenecks than is the diversity of the mitochondrial genome or the haploid Y chromosome. For example, a Finnish bottleneck that left a strong imprint on Y chromosome diversity led to no reduction in autosomal diversity [28]. Recently, Kanthaswamy et al. reported that Chinese and Indian macaques appeared to have nearly identical genetic diversity based on genotype analysis with more than 1,000 SNPs [11]. It should be noted that the similarity of diversity of the one Chinese and two Chinese/Indian admixed macaques we studied does not address whether there are significant differences at the haplotype level. Deeper insight into haplotype structures and diversity in different species of non-human primates remains an important future direction, requiring analyses to include multiple individuals from different subpopulations.

There is some evidence that the mutation rate may have slowed in the hominoid ape lineage, but based on the nucleotide diversity rates we have observed, we can compare the effective population sizes of rhesus macaque and human. For this purpose, we used Watterson’s (1975) [29] estimator θ = 4*N*e*u* with average nucleotide diversity (θ_SNP_) in intergenic regions (Figure 2C) as θ because intergenic diversity is most likely to faithfully reflect neutral diversity at the whole genome level. Assuming an average mutation rate of 1 x 10^-8^ to 2.5 x 10^-8^ mutations per nucleotide site per diploid genome per generation for human [17,30-32] and an average mutation rate 5.9 x 10^-9^ mutations per nucleotide site per diploid genome per generation for macaque [15], the effective population size of humans is approximately 6,780-16,950 and the effective population size of the macaque is approximately 80,000. The effective population size we have estimated for macaques may not obtain for animals from subpopulations which we have not sampled, particularly in the case of Chinese macaques. In our study, most powerful comparison is the diversity ratio of the human versus the rhesus macaque, with the macaque emerging as having an effective population size many times larger.

From our comparative analysis of large number of SNPs equivalently identified from 14 humans and 14 rhesus macaques, the rhesus macaque has three times the SNP density and nucleotide diversity of humans. However, macaque has a lower ratio of nonsynonymous to synonymous substitutions in cSNPs, and the total number of damaging coding variations in individual macaques is closely equivalent to the human, indicating that selection has dampened the increase in functional diversity in this species.

## Conclusions

Using an equivalent methodology and sampling space for the human and rhesus macaque, we identified 462,802 macaque SNPs, most novel and disproportionately located in functionally important genomic regions. Accuracy of macaque SNP identification was conservatively estimated to be >90% based on verification with Sanger sequencing and because 90% of SNPs identified in our human samples using parallel methods were previously listed in dbSNP. This large panel of newly identified macaque SNPs significantly expands our knowledge of genetic variation in the rhesus macaque. Comparative analysis with the human reveals that this widespread, highly adaptable species is approximately three times as diverse as the human but more closely equivalent in damaging variation.

## Methods

### Samples and tissues

Postmortem brain tissue (hippocampus) of 14 unrelated human (*H. sapiens*) males, age 30–50 yrs was obtained from the University of Miami Brain Endowment Bank (Miami, FL, USA). In ethnic background the sample consisted of six African Americans and eight Caucasians/Hispanics. Postmortem hippocampus of 14 rhesus macaque (*M. mulatta*) males, most unrelated, age 3.5-7 yrs, was obtained from the National Institutes of Health Animal Center in Poolesville, Maryland. Among the macaques, eleven were of Indian origin, one was of Chinese origin and two were approximately 50% Chinese/50% Indian as indicated by genotyping of a panel of 96 markers, which have been used to screen over 600 rhesus macaques for ancestry determination optimized for macaque population origin (source: Primate Genetics Program, Oregon National Primate Research Center, Additional file 1 Table S1) [33]. The macaques at the Poolesville colony are maintained in an outbred state, with frequent introduction of new breeding stock such that their genetic diversity is expected to be equivalent to natural populations. The human research protocol for procedures involved in collecting postmortem brain and associated data was approved by the University of Miami. Animal study protocols were approved by the NIAAA and NICHD Animal Care and Use Committees.

### Construction of double-stranded cDNA libraries

Total RNA was extracted from 100 mg of hippocampus collected postmortem. Briefly, tissue samples were submerged in guanidinium thiocyanate and phenol-based RNA extraction solution STAT-60 (Tel-Test Inc.*,* Friendswood, TX) and homogenized using a glass-Teflon homogenizer. Following mixing with chloroform and centrifugation, the aqueous phase was collected and isopropanol was added. The samples were then loaded onto RNeasy spin columns (Qiagen, Valencia, CA) for purification. To eliminate residual genomic DNA contamination, RNA samples were incubated with DNase I (Qiagen) on column at room temperature for 15 min and washed several times before collection in elution buffer. To isolate mRNA, 35 μg of total RNA was heated at 65°C for 2 min, and then mixed with 0.5 mg of Dynabeads oligo (dT)_25_ (Invitrogen, Carlsbad, CA) in binding buffer (20 mM Tris–HCl, pH 7.5, 1.0 M LiCl, 2 mM EDTA). After incubation at room temperature for 5 min and several washing steps, mRNA was eluted from the beads by heating at 80°C for 2 min. The purified mRNA was fragmented to the 150 – 500 bp range by mixing with 10x fragmentation buffer (Ambion, Austin, TX) and heating at 70°C for 3 min. The samples were purified with RNeasy spin column. Two hundred nanograms of fragmented mRNA was reverse-transcribed to first strand cDNA by random priming, using 3 μg of random hexamer oligos and 200 units of Superscript II reverse transcriptase (Invitrogen). The reaction was carried out at 45°C for 1 hr in First Strand Buffer (Invitrogen) with 10 mM DTT and 0.5 mM dNTP. For second-strand cDNA synthesis, 400 units of *Escherichia coli* DNA polymerase, 2 units of *E. coli* RNase H, and 10 units of *E. coli* DNA ligase was added, and the reaction was carried out at 16°C for 2 hr in Second Strand Buffer with 0.2 mM dNTP. Twenty units of T4 DNA polymerase was also added at the end of incubation for end repair. The synthesized double-stranded cDNA library was purified with QIAquick purification kit (Qiagen).

### Chromatin immunoprecipitation (ChIP)

Postmortem brain tissue (100 mg) was cut into slices less than 1 mm in thickness, and fixed in 3 ml of 1% formaldehyde/PBS solution for 10 min at room temperature to cross-link chromatin DNA and proteins. The tissue samples were then homogenized using a glass-Teflon homogenizer. Following homogenization, chromatin was isolated using the Upstate Magna ChIP G kit (Millipore, Temecula, CA). Briefly, cells were lysed in Cell Lysis Buffer in the presence of protein inhibitor cocktail. Nuclei were isolated from lysed cells by centrifugation, and re-suspended in Nuclear Lysis Buffer. The chromatin DNA was then fragmented into the 150 – 500 base-pair range by sonication using a Branson Sonifer (Branson, Danbury, CT). To immunoprecipitate specific genomic regions of chromatin DNA, isolated chromatin was incubated with antibodies (Abcam, Cambridge, MA) against H3K4me3 and magnetic protein G beads (Millipore) at 4°C for 2.5 hr. Following incubation, beads were washed with low salt, high salt, LiCl salt, and TE buffers, and reverse cross-linked by proteinase K digestion at 62°C for 2 hr. The enriched DNA was purified after reverse cross-linking by column purification.

### Sequencing with Illumina Genome Analyzer

Sample preparation and sequencing on an Illumina Genome Analyzer (Illumina, San Diego, CA) were carried out according to Illumina protocols for single-end libraries with some modifications. Briefly, double-stranded cDNA and ChIP-enriched genomic DNA were treated with T4 DNA polymerase and Klenow fragment for end repair. The 5’ ends of DNA fragments were then phosphorylated by T4 polynucleotide kinase, and an adenosine base was added to the 3’ end of the fragments by Klenow (3’-5’ exo^-^). A universal adaptor was added to the both ends of the DNA fragments by A-T ligation. Following 18 cycles of PCR with Phusion DNA polymerase, the DNA library was purified on a 2% agarose gel, and fragments 170 – 350 bp in size were recovered. Approximately 10 ng of the prepared DNA was then used for cluster generation on a grafted Flow Cell, and sequenced on the Genome Analyzer for 36 cycles using the “sequencing-by-synthesis” method.

### SNP calling and sequence analyses

Sequences were called from image files with the Illumina Genome Analyzer Pipeline (GApipeline) and aligned to the corresponding reference genome (UCSC rheMac2 for macaque and UCSC hg18 for human) using Extended Eland in the GApipeline. The uniquely mapped reads were parsed with in-house Perl scripts to generate base coverage and SNP calls as described previously [18]. To reduce false positive and false negative SNP calling for low coverage sequence data, a two-step approach was used. Briefly, reads were first pooled from all samples in a species for SNP identification. At this step, no base in the uniquely mapped reads had a quality score < 8, only a single mis-match with quality score > = 15 was allowed in a single 36-base read, and a probable SNP had to have three independent reads representing the same alternative allele within the pooled samples. To reduce false SNP calls due to mis-mapping of cross-exon RNAseq reads, putative SNPs were filtered to remove instances in which the alternative allele was represented only by reads located one or two bases from either end of the RNAseq fragment. Candidate SNPs were then filtered at the individual sample level, where the frequency of the alternative allele in a single sample had to be the highest or second highest with a frequency ≥ 0.2. Genotypes were called for an individual sample only when sequencing coverage was ≥ 6x for the SNP site and when the allele with the lowest coverage was represented at least 3 times and heterozygotes with each allele covered by 30 ~ 70% of sequence reads. Gene structures for human were based on RefSeq Genes in UCSC hg18 and Ensembl Genes from UCSC rheMac2 were used for the macaque. PolyPhen-2 [34] was used to predict protein functional effects of nonsynonymous coding SNPs (nsSNPs). Fourteen novel macaque cSNPs were selected to be resequenced by Sanger sequencing using the BigDye Terminator Sequencing Mix (Applied Biosystems, Carlsbad, CA) and analyzed on the Applied Biosystems 3730 DNA Analyzer.

## Authors’ contributions

DG, QY and CB conceived this study. ZZ performed all next-generation sequencing. QY analyzed sequencing data. QY and DG prepared the manuscript. BB performed SNP functional predictions. SL and MB performed SNP verification using Sanger sequencing. DM provided human postmortem samples and perspective on the project design. DH, BF, and SS provided leadership and insights in rhesus macaque biology. DH and SS provided essential input in the population history of the Poolesville colony and played leadership roles in the creation and maintenance of that colony. JL and RT collected and dissected the brain tissue. BF was the source of the macaque population SNP panel. All authors read and approved this manuscript.

## Supplementary Material

Additional file 1 **Table S1.** Ancestry assay for rhesus macaque samples used in this study*. **Table S2.** Putative SNPs covered with sequence reads from ChIPseq and/or RNAseq. **Table S3.** SNPs from 1000 Genomes Project Pilot 2. Table S4. The average nucleotide heterozygosity from 1000 Genomes Project Pilot 2. **Figure S1:** A: RNAseq correlation between human vs macaque. Data points: mean of normalized gene expression level (log2). B: H3K4me3 ChIPseq correlation between human vs macaque. Data points mean of normalized area under curve (log10) of covered reads within 1Kb of TSS. C. Sequencing coverage (H3K4me3 ChIPseq and RNAseq) in NPY genic region. **Figure S2:** Human cSNPs identified in 1000 Genomes Project Pilot 2 samples and this study. C or CEU: CEU trio from 1000 Genomes Project Pilot 2; Y or YRI: YRI trio from 1000 Genomes Project Pilot 2; M or Miami: 14 samples from Miami dataset in this study. **Figure S3:** SNPs shared between individuals in 1000 Genomes Project Pilot 2. Click here for file
