# Supplementary material for: The rhesus macaque is three times as diverse but more closely equivalent in damaging coding variation as compared to the human
Source: BMC Genet. 2012 Jun 29;13:52. doi: 10.1186/1471-2156-13-52 (PMC3426462; doi:10.1186/1471-2156-13-52)
Supplement: Additional file 1 — Table S1. Ancestry assay for rhesus macaque samples used in this study*. Table S2. Putative SNPs covered with sequence reads from ChIPseq and/or RNAseq. Table S3. SNPs from 1000 Genomes Project Pilot 2. Table S4. The average nucleotide heterozygosity from 1000 Genomes Project Pilot 2. Figure S1: A: RNAseq correlation between human vs macaque. Data points: mean of normalized gene expression level (log2). B: H3K4me3 ChIPseq correlation between human vs macaque. Data points mean of normalized area under curve (log10) of covered reads within 1Kb of TSS. C. Sequencing coverage (H3K4me3 ChIPseq and RNAseq) in NPY genic region. Figure S2: Human cSNPs identified in 1000 Genomes Project Pilot 2 samples and this study. C or CEU: CEU trio from 1000 Genomes Project Pilot 2; Y or YRI: YRI trio from 1000 Genomes Project Pilot 2; M or Miami: 14 samples from Miami dataset in this study. Figure S3: SNPs shared between individuals in 1000 Genomes Project Pilot 2. [file 1471-2156-13-52-S1.doc]

Table S1. Ancestry assay for rhesus macaque samples used in this study*

| SampleID | %MissingData | Inferred cluster | | 90% Probability Interval | |
| --- | --- | --- | --- | --- | --- |
|  |  | Chinese | Indian | Chinese | Indian |
| E13 | 0 | 0.006 | 0.994 | (0.000,0.044) | (0.956,1.000) |
| E20 | 0 | 0.007 | 0.993 | (0.000,0.046) | (0.954,1.000) |
| E78 | 0 | 0.005 | 0.995 | (0.000,0.034) | (0.966,1.000) |
| E85 | 0 | 0.524 | 0.476 | (0.392,0.653) | (0.347,0.608) |
| G14 | 0 | 0.013 | 0.987 | (0.000,0.087) | (0.913,1.000) |
| G36 | 0 | 0.531 | 0.469 | (0.395,0.664) | (0.336,0.605) |
| K09 | 0 | 0.004 | 0.996 | (0.000,0.027) | (0.973,1.000) |
| K20 | 0 | 0.999 | 0.001 | (0.991,1.000) | (0.000,0.009) |
| K29 | 0 | 0.003 | 0.997 | (0.000,0.018) | (0.982,1.000) |
| K41 | 0 | 0.008 | 0.992 | (0.000,0.052) | (0.948,1.000) |
| K44 | 0 | 0.006 | 0.994 | (0.000,0.043) | (0.957,1.000) |
| K46 | 0 | 0.005 | 0.995 | (0.000,0.034) | (0.966,1.000) |
| M18 | 0 | 0.019 | 0.981 | (0.000,0.102) | (0.898,1.000) |
| M40 | 0 | 0.004 | 0.996 | (0.000,0.027) | (0.973,1.000) |

* The rhesus macaque SNP ancestry assay included 96 independent polymorphic loci that were previously selected based upon their differential allele frequencies in a survey of known Indian and Chinese rhesus macaques. DNAs from the animals in this study were genotyped using the 96 SNP ancestry panel, along with control subjects with known Indian and Chinese ancestry, using a custom Illumina Goldengate SNP assay. The resulting genotypes were analyzed using STRUCTURE software (Pritchard et al., 2000, Inference of population structure using multilocus genotype data, Genetics 155:945-959) to discern the 90% credible interval membership of each population for each individual. Individuals for which the Indian rhesus contribution fell below a possible 100% membership were identified.

Table S2. Putative SNPs covered with sequence reads from ChIPseq and/or RNAseq

|  | **Human SNPs having reads from** | | | | **Macaque SNPs having reads from** | | | |
| --- | --- | --- | --- | --- | --- | --- | --- | --- |
|  | **ChIPseq** | **RNAseq** | **Both** | **Total** | **ChIPseq** | **RNAseq** | **Both** | **Total** |
| **Total** | 130914 | 35615 | 63499 | 230028 | 386219 | 23109 | 53474 | 462802 |
| **Intergenic** | 80482 | 11594 | 15385 | 107461 | 240655 | 9457 | 19278 | 269390 |
| **5Kbupstream** | 7404 | 131 | 2501 | 10036 | 22057 | 403 | 3843 | 26303 |
| **Intron** | 39458 | 8736 | 31681 | 79875 | 113994 | 2267 | 14182 | 130443 |
| **Exon** | 3570 | 15154 | 13932 | 32656 | 9513 | 10982 | 16171 | 36666 |
| **UTR** | 1966 | 9149 | 7317 | 18432 | 4249 | 4815 | 6391 | 15455 |
| **CDS** | 1604 | 6005 | 6615 | 14224 | 5264 | 6167 | 9780 | 21211 |
| **nsSNP** | 814 | 2513 | 2550 | 5877 | 2453 | 1864 | 3050 | 7367 |
| **synonymous** | 787 | 3485 | 4057 | 8329 | 2781 | 4299 | 6718 | 13798 |
| **nonsense** | 3 | 7 | 8 | 18 | 30 | 4 | 12 | 46 |

Table S3. SNPs from 1000 Genomes Project Pilot 2

|  | CEU.trio | YRI.trio |
| --- | --- | --- |
| Total | 3646764 | 4502439 |
| Also in dbSNP | 3239544(88.8%) | 3446643(76.6%) |
| Located in intergenic | 2131596(58.4%) | 2599459(57.7%) |
| Located in 5Kb upstream | 165384(4.5%) | 210006(4.7%) |
| Located in intron | 1336273(36.6%) | 1674001(37.2%) |
| Located in UTR | 31887(0.9%) | 41315(0.9%) |
| Located in CDS | 24192(0.7%) | 32244(0.7%) |
| nsSNP | 9696(40.1%) | 12853(39.9%) |
| Synonymous | 14506(60.0%) | 19412(60.2%) |

Table S4. The average nucleotide heterozygosity from 1000 Genomes Project Pilot 2

| Population | SampleID | θ_SNP_ (x10^-4^)* |
| --- | --- | --- |
| CEU | NA12891 | 7.16 |
| CEU | NA12892 | 7.33 |
| YRI | NA19239 | 9.26 |
| YRI | NA19238 | 9.06 |

*: Calculated as the heterozygous bases divided by sequencing accessible genome size (2.85 x 10^9^ x 80%) using 1000 Genomes Project Pilot 2 data.

Fig. S1: A: RNAseq correlation between human vs macaque. Data points: mean of normalized gene expression level (log2). B: H3K4me3 ChIPseq correlation between human vs macaque. Data points mean of normalized area under curve (log10) of covered reads within 1Kb of TSS. C. Sequencing coverage (H3K4me3 ChIPseq and RNAseq) in NPY genic region.

Fig. S2: Human cSNPs identified in 1000 Genomes Project Pilot 2 samples and this study. C or CEU: CEU trio from 1000 Genomes Project Pilot 2; Y or YRI: YRI trio from 1000 Genomes Project Pilot 2; M or Miami: 14 samples from Miami dataset in this study.

Fig. S3: SNPs shared between individuals in 1000 Genomes Project Pilot 2.
